# Cleansing effect of acidic L-arginine on human oral biofilm

**DOI:** 10.1186/s12903-016-0194-z

**Published:** 2016-03-22

**Authors:** Ayano Tada, Haruyuki Nakayama-Imaohji, Hisashi Yamasaki, Khaleque Hasibul, Saori Yoneda, Keiko Uchida, Hirofumi Nariya, Motoo Suzuki, Minoru Miyake, Tomomi Kuwahara

**Affiliations:** Department of Microbiology, Faculty of Medicine, Kagawa University, 1750-1, Miki, Kagawa 761-0793 Japan; Department of Dental Oral Surgery, Faculty of Medicine, Kagawa University, 1750-1, Miki, Kagawa 761-0793 Japan; Department of Cellular and Molecular Medicine, Wakayama Medical University Graduate School of Medicine, Wakayama, 641-8509 Japan

**Keywords:** L-arginine, Biofilm, Mouth rinse, Oral microbiome, Saliva

## Abstract

**Background:**

Dental plaque formed on tooth surfaces is a complex ecosystem composed of diverse oral bacteria and salivary components. Accumulation of dental plaque is a risk factor for dental caries and periodontal diseases. L-arginine has been reported to decrease the risk for dental caries by elevating plaque pH through the activity of arginine deiminase in oral bacteria. Here we evaluated the potential of L-arginine to remove established oral biofilms.

**Methods:**

Biofilms were formed using human saliva mixed with Brain Heart Infusion broth supplemented with 1 % sucrose in multi-well plates or on plastic discs. After washing the biofilms with saline, citrate (10 mM, pH3.5), or L-arginine (0.5 M, pH3.5), the retained biofilms were analyzed by crystal violet staining, scanning electron microscopy, and Illumina-based 16S rDNA sequencing.

**Results:**

Washing with acidic L-arginine detached oral biofilms more efficiently than saline and significantly reduced biofilm mass retained in multi-well plates or on plastic discs. Illumina-based microbiota analysis showed that citrate (pH3.5) preferentially washed out *Streptococcus* from mature oral biofilm, whereas acidic L-arginine prepared with 10 mM citrate buffer (pH3.5) non-specifically removed microbial components of the oral biofilm.

**Conclusions:**

Acidic L-arginine prepared with citrate buffer (pH3.5) effectively destabilized and removed mature oral biofilms. The acidic L-arginine solution described here could be used as an additive that enhances the efficacy of mouth rinses used in oral hygiene.

**Electronic supplementary material:**

The online version of this article (doi:10.1186/s12903-016-0194-z) contains supplementary material, which is available to authorized users.

## Background

There is accumulating evidence that deterioration of oral hygiene is associated with periodontal disease [1] as well as increased risk for cardiovascular disease [2, 3], metabolic syndrome [4, 5], and pre-term birth [6]. Oral hygiene care is also recognized as a critical measure that reduces the risks for health care-associated pneumonia (HCAP). Oral biofilms can act as reservoirs for pneumonia-related bacteria, as suggested by the similarity between oral microbiota and clinical HCAP samples [7–9]. Many clinical trials that investigated the effectiveness of oral hygiene care on prophylaxis for HCAP, including pneumonia arising from mechanical ventilation, have been reported. These trials found: (i) mechanical and chemical oral hygiene measures reduced the rate of respiratory pathogen colonization in oral microbiota [10, 11]; (ii) oral hygiene care with either chlorhexidine mouthwash or gel was associated with a 40 % reduction in ventilator-associated pneumonia in critically ill adults [12]; and (iii) mechanical oral cleaning significantly reduced the risk for fatal pneumonia [13].

Oral cavities contain diverse microbiota that numbers nearly 700 species [14]. This ecosystem includes biofilm-forming bacteria such as *Streptococcus mutans*, *Fusobacterium nucleatum*, *Porphyromonas gingivalis*, and *Aggregatibacter actinomycetemcomitans*. Although the microbiota varies among individuals, within a given individual the oral microbiome is relatively stable [15]. However, the oral microbial composition can change rapidly under compromised conditions that can occur during hospitalizations [16]. Therefore, oral hygiene care for critically ill patients is especially important to prevent colonization of multi-drug resistant bacteria or overgrowth of respiratory pathogens. Inadequate oral hygiene may result in the formation of mature dental plaque on tooth surfaces or oral epithelial cells, and this plaque can be resistant to cleansing by rinsing with water or even with manual tooth brushing, which highlights the need for effective strategies that suppress oral biofilm formation. Various trials to test the ability of herbal extracts [17], disinfectants [18, 19], inhibitors of polysaccharide production [20, 21], and rare sugars [22] to reduce formation of oral biofilms have been conducted.

Recently, alkali-generating agents such as L-arginine and urea were predicted to inhibit the growth of acidogenic or aciduric bacteria by raising the pH of the oral environment [23]. Metabolism of L-arginine and urea by arginine deiminase and urease, respectively, produces ammonia. Many oral bacteria possess an arginine deiminase system, which metabolizes L-arginine to produce ornithine and ammonia that increases the pH of oral biofilms. Clinical trials investigating L-arginine-containing dentifrices or mouth rinses showed a protective effect against development of dental caries in young subjects [24, 25]. In addition, L-arginine and fluoride synergistically suppressed *S. mutans* growth and biofilm formation [26].

L-arginine is a basic amino acid that contains a guanidine group. Like guanidine hydrochloride, L-arginine can increase protein solubility and suppress protein aggregation [27]. Although the precise mechanism for L-arginine-mediated inhibition of protein-protein interactions remains unclear, L-arginine can change the surface tensions of proteins by interacting with proteins or the water surrounding them without the tight attachment seen with other agents such as guanidine hydrochloride [28]. Because this mild interaction preserves protein functions, L-arginine has been used for solubilization of exogenously expressed proteins or antibodies for pharmaceutical use. In addition, L-arginine was recently reported to diminish the infectivity of envelope viruses such as herpes simplex virus and influenza virus, probably due to compromised function of proteins in the envelope [29].

Oral biofilm is a complex ecosystem that is composed of diverse bacteria, insoluble glucan, and salivary glycoproteins. Removing this solid biological plaque from tooth surfaces by simple water rinsing or even with mechanical brushing can be difficult. Residual plaque also serves as a base for further biofilm formation. Because L-arginine is expected to inhibit oral biofilm formation and also destabilize complex aggregates in dental plaque, inclusion of this amino acid in a mouth rinse could facilitate oral biofilm removal. In this study, we evaluated the potential of L-arginine as a cleanser to remove already established oral biofilms.

## Methods

### Killing assay for *Streptococcus mutans*

Glycerol stocks of *S. mutans* GS5 were streaked on Brain Heart Infusion (BHI) agar plates that were then incubated anaerobically at 37 °C for 48 h. Anaerobic culture was performed using an AnaeroPack system (Mitsubishi Gas Co., Ltd.). Several *S. mutans* GS5 colonies were inoculated into BHI broth and cultivated at 37 °C for 48 h. The cultures were centrifuged at 15,000 rpm for 5 min and resuspended in saline. Bacterial suspensions (0.1 ml) were added to 0.9 ml of saline, 10 mM citrate (pH3.5), or 0.5 M L-arginine in 10 mM citrate (pH3.5). After incubating for 5 min at 37 °C, serial 10-fold dilutions with phosphate-buffered saline (PBS, pH7.4) were prepared, and 0.1 ml of the appropriate dilution was spread onto BHI agar plates. After a 72 h anaerobic incubation at 37 °C, the number of colonies was counted and compared with the number in the saline treatment.

### Biofilm inhibition assay

To assess the inhibitory effect on *S. mutans* GS5 biofilm formation, 0.1 ml saline, 10 mM citrate (pH3.5), or 0.5 M L-arginine pH3.5–7.0 (adjusted by 10 mM citrate buffer) were mixed with an equal volume of the bacterial suspension (1 % v/v of 48 h culture) in 2 x BHI containing 2 % sucrose. The mixtures were added to 96-well plates and incubated anaerobically at 37 °C. After a 24 h cultivation, the biofilms formed on the well bottoms were quantified by crystal violet staining as described below.

### Collection of human salivary samples

After written informed consent was obtained from nine healthy volunteers (21–27 years old, male), they were requested to collect saliva excreted while chewing wax gum for 5 min. Exclusion criteria were younger than 20 years old or having received antibiotic treatment within the previous 4 weeks.

### Cleansing effect of L-arginine on oral biofilm formed on plastic discs

Human oral biofilms were formed on 13.5-mm sterile plastic discs (Sensi-Disc, Sumitomo Bakelite, Co., Ltd., Tokyo), set in 24-well culture dishes. The discs were incubated in 0.5 ml BHI containing 1 % sucrose and 20 % human saliva collected from three healthy volunteers (#1-#3). After a 72 h anaerobic cultivation, the discs were removed and washed once with saline. The biofilms on the discs were further washed with 0.5 ml of 10 mM citrate (pH3.5), 0.5 M each of L-arginine, L-alanine, L-glycine or L-lysine (all adjusted the pH at 3.5 by 10 mM citrate) by shaking for 30 min at 37 °C. The discs were then washed again with 0.5 ml PBS (pH7.4), stained with 0.5 ml 0.01 % crystal violet for 20 min at room temperature. The discs were then washed four times with 1.0 ml saline and air-dried. After taking photograph, the remaining dye was eluted with 0.5 ml 33 % acetic acid by shaking for 30 min at room temperature. The absorbance of the eluents at 550 nm was then measured.

### Cleansing assay on human salivary biofilm using microwells

Human saliva collected from four healthy volunteers (#4-#7) were added to BHI containing 1 % sucrose at 20 % (v/v). Modified human oral biofilms were also prepared by adding 48 h *S. mutans* GS5 cultures (1 % final volume) to simulate cariogenic biofilm. Each mixture (0.1 ml) was then added to 96-well plates and incubated anaerobically at 37 °C. After 72 h, the cultures were removed, and the biofilm formed on the bottom of the plates was washed once with 0.2 ml saline. Each well was then washed with 0.1 ml saline (control), 10 mM citrate (pH3.5, solvent for L-arginine in this study), or 0.5 M L-arginine (pH3.5) by shaking for 30 min at 37 °C. The test reagents were decanted and each well was washed three times with 0.2 ml saline. The remaining biofilm was stained with 0.1 ml 0.01 % crystal violet for 20 min at room temperature. After staining, the wells were washed four times with 0.2 ml saline, and the remaining dye was eluted with 0.2 ml 33 % acetic acid by shaking for 30 min at room temperature. The absorbance of the eluents at 550 nm was then measured. Six wells were used for each sample in a single assay. Assays were repeated three times independently.

### Scanning electron microscopy

Human oral biofilms were formed on 13.5-mm sterile plastic discs using the samples from #4 to #7. The discs were incubated in 0.5 ml BHI containing 1 % sucrose and 20 % human saliva. After a 72 h anaerobic cultivation, the discs were removed and washed once with saline. The biofilms on the discs were further washed with 0.5 ml saline, 10 mM citrate (pH3.5), or 0.5 M L-arginine (pH3.5) by shaking for 30 min at 37 °C. The discs were then washed again with 0.5 ml PBS (pH7.4), and the biofilms on the discs were fixed with 2 % glutaraldehyde in 0.1 M cacodylate buffer (pH7.2). After fixation, the biofilms were dehydrated in a graded ethanol series and dried in a Hitachi PCP-2 critical point drying apparatus. The discs were coated with platinum/palladium in a Hitachi E-102 sputter coater and examined with a JEOL JCM-6000 scanning electron microscope. The biofilm area retained after washing with each test reagent was measured using the color auto-selection tool in Photoshop CS6. The pixel value ratios of the biofilm area to total observation area were calculated from five randomly selected fields at 100x magnification.

### Microbial composition analysis by 16S rDNA sequencing

The salivary microbiome of the six healthy volunteers (#4-#9) was characterized by Illumina Miseq 16S rDNA sequencing analysis of DNA extracted from 3 ml saliva. To identify the microbial groups that were sensitive to citrate or L-arginine rinsing, human saliva-derived biofilms formed on 13.5-mm plastic discs were washed with the test reagents described above. The test reagents were then recovered, and the bacteria from the washings were collected by centrifugation (washed-out fraction). The discs were washed three times with 0.5 ml saline, cut into pieces with sterile scissors, and then transferred to 0.5 ml saline for sonication with a Bioruptor (Cosmobio, 3 x 1 min with 1-min interval, output setting H). The detached biofilms were collected by centrifugation (sustained fraction). DNA from both fractions was also purified and the microbial composition determined by 16S rDNA sequencing analysis.

DNA extraction was performed according to the method reported by Morita et al. [30]. Sequencing libraries were prepared by amplifying the V3-V4 region of the 16S rDNA using the primers described by Klindworth et al. [31]. After initial amplification, a second PCR was performed to attach Illumina adaptors as well as barcodes that allowed for multiplexing. Amplifications were performed in 25 μl reactions containing 2.5 μl diluted template, 12.5 μl 2x KAPA HiFi HotStart Ready Mix and 2.5 μl of each primer. Thermal cycling consisted of an initial denaturation step (3 min at 95 °C), followed by 25 cycles of denaturation (30 s at 95 °C), annealing (30 s at 55 °C) and 30 s extension at 72 °C. The final extension step consisted of 5 min at 72 °C. Amplicons were purified using AMPure XP beads (Beckman Coulter). Sequencing was performed on the Illumina MiSeq platform (MiSeq Reagent Kit ver. 3, 600 cycles) according to the manufacturer’s specifications to generate paired-end reads of 300 bases in each direction. A total of 10,381,210 2 x 300 base pair reads with an average of 432,550 reads per sample was obtained. Primer sequences were trimmed, and the paired-end reads merged using Fastq-join [32] with default parameters and processed with the QIIME 1.8.0 pipeline [33]. After a chimera check by Usearch, 20,000 Illumina reads per sample (average quality score above 20) were randomly selected for further analysis. Using the UCLUST [34] algorithm built into the QIIME pipeline, sequences were clustered at > 97 % identity against the Greengenes reference database, producing 635 operational taxonomic units (OTUs). Using the QIIME pipeline, unweighted UniFrac distances were produced and used to investigate beta diversity by plotting PCA coordinates.

### Nucleotide seqeunce

Sequence data have been deposited in DDBJ database (accession number: DRA004109, PRJDB4298, SAMD00042426-SAMD00042441).

### Statistics

Statistical analysis of the data was performed with StatFlex ver. 6.0 (Artech Co., Ltd., Tokyo) using analysis of variance (ANOVA) to compare the means of all groups and followed by Tukey’s test to compare the means of each of the two groups. Data were considered to be significantly different if the 2-tailed *p* value was less than 0.05.

### Ethics consent and permissions

Human saliva were collected from nine healthy volunteers after written informed consent was obtained. Ethical approval was obtained from the Research Ethics Committee of the Faculty of Medicine, Kagawa University (reference number, 27-074).

### Consent to publish

The authors obtained consent from all of the participants to publish the analysis data under linkable anonymizing.

## Results

### Inhibitory effect of L-arginine on *S. mutans* GS5 biofilm formation

**Fig. 1:**
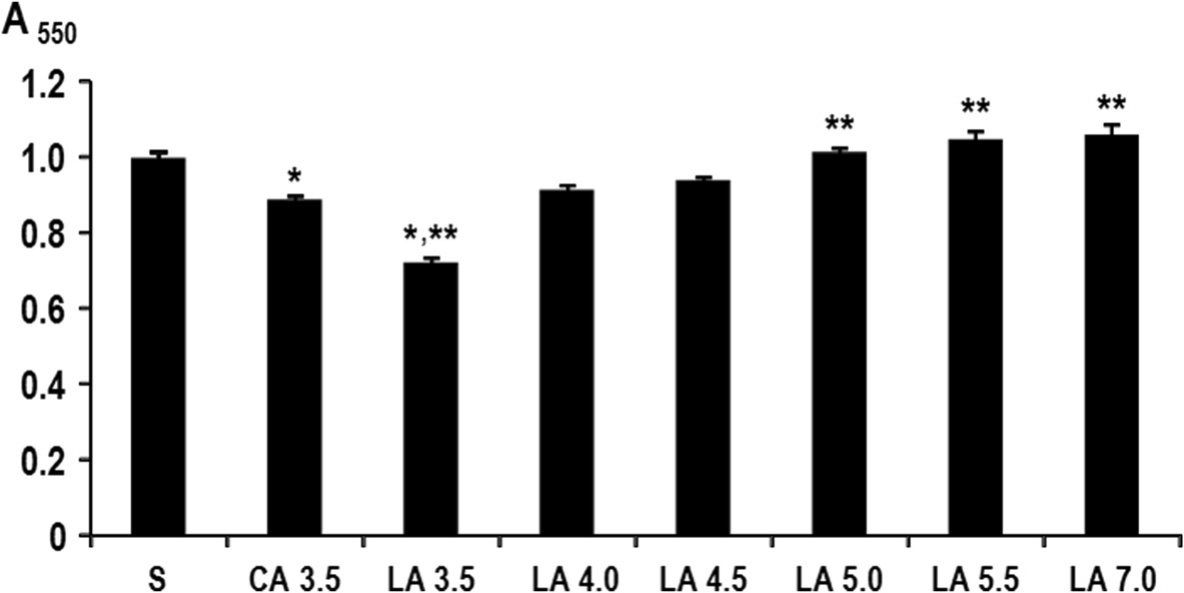
pH-dependent inhibition of *S. mutans* GS5 biofilm formation by L-arginine. Diluted *S. mutans* GS5 cultures and test reagents were mixed at 1:1. After the mixtures were cultured anaerobically at 37 °C for 24 h, *S. mutans* biofilms that had formed at the bottom of the microwells were quantified by crystal violet staining. Saline (S), 10 mM citrate pH3.5 (CA3.5) or 0.5 M L-arginine solution adjusted with 10 mM citrate buffer to a pH between 3.5 and 7.0 (indicated as LA3.5-LA7.0) were used as test reagents. The data are expressed as means ± standard deviation. ^*^ Significantly different from saline (*p* < 0.05), ^**^ Significantly different from 10 mM citrate (pH3.5) (*p* < 0.05)

Biofilm assays were performed to determine the effect of L-arginine on *S. mutans* GS5 biofilm formation under pH conditions ranging from acidic to neutral (pH3.5–7.0). Firstly, *S. mutans* GS5 culture was added to 2 x BHI containing 2 % sucrose at 2 % (v/v). The bacterial suspension (0.1 ml) was then mixed with saline, 10 mM citrate (pH3.5) or 0.5 M each of L-arginine solution adjusted its pH (3.5–7.0) and applied to the microwells. After 24-h anaerobic cultivation, *S. mutans* biofilm was quantified by crystal violet staining. As shown in Fig. 1, L-arginine solutions (final concentration, 0.25 M) that were adjusted to pH3.5 significantly inhibited *S. mutans* biofilm after 24 h of cultivation when compared with saline or buffer control (5 mM citrate, pH3.5). The effect of L-arginine was pH-dependent, wherein only L-arginine solutions adjusted to pH3.5 significantly inhibited biofilm formation. Furthermore, this inhibition was unlikely to be due to a bactericidal effect, because 10 mM citrate buffer (pH3.5) and L-arginine adjusted to pH3.5 reduced *S. mutans* GS5 number by only 0.61 ± 0.30 and 0.49 ± 0.22 log_10_ CFU/ml during a 5-min contact, respectively, and the optical density of the *S. mutans* culture after 24 h incubation was not different among the samples with and without 0.25 M L-arginine solution (pH3.5). These results suggest that acidic L-arginine destabilized biofilm structure and/or reduced insoluble glucan production of *S. mutans*. Based on these results, acidic L-arginine solutions adjusted to pH3.5 were used for subsequent analyses.

### Cleansing effect of L-amino acid solution on oral biofilm

**Fig. 2:**
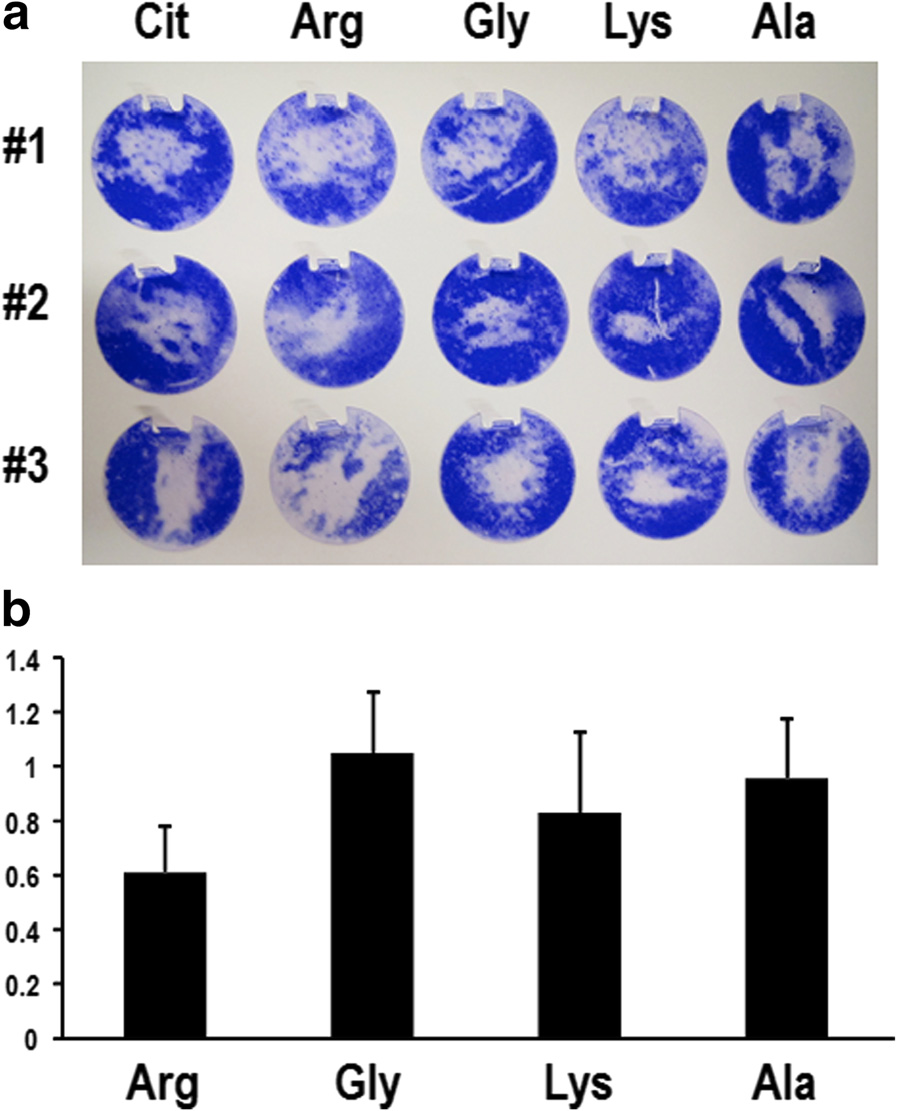
Cleansing effect of L-arginine on the salivary biofilm formed on plastic discs. **a** Crystal violet staining of the biofilm retaining on the discs after washing. The biofilm formed on the plastic disc was washed for 30 min with 10 mM citrate buffer (pH3.5; Cit), 0.5 M each of L-arginine (Arg), L-glycine (Gly), L-lysine (Lys) or L-alanine (Ala), which were adjusted the pH to 3.5 with 10 mM citrate buffer. **b** Quantification of the salivary biofilm retaining on the discs. The crystal violet was eluted from the discs shown in panel A with acetic acid and their absorbance at 550 nm was measured. Relative values to citrate sample are shown

Saliva-derived biofilm was prepared on plastic discs using the saliva samples from healthy adults (#1, #2, and #3). After the biofilm was washed with 10 mM citrate (pH3.5), 0.5 M each of L-arginine, L-glycine, L-lysine or L-alanine solutions, which were adjusted the pH to 3.5. As shown in Fig. 2a, the crystal violet stains on the discs washed with L-arginine were less than those washed with citrate buffer or the other three L-amino acids tested. The crystal violet dyes were eluted with acetic acid, and the absorbance at 550 nm of the elution was measured to quantify the retaining biofilms on the discs. As shown in Fig. 2b, L-arginine reduced human salivary biofilm more effectively than other L-amino acids although significant difference was not observed.

### Destabilizing effect of acidic L-arginine on oral biofilms

To further evaluate the destabilization effect of acidic L-arginine (pH3.5) on human oral biofilm, the saliva samples were collected from four healthy volunteers (#4, #5, #6, and #7). Illumina-based 16S rDNA sequencing analysis showed that these samples contained the microbiome consistent with several previous reports on human saliva [35, 36]: *Streptococcus* (most predominant, 23.2–44.5 % abundance rate in this study), *Prevotella*, *Neisseria*, *Veillonella*, *Rothia*, *Fusobacterium*, *Gemella*, *Porphyromonas*, *Haemophilus*, *Granulicatella,* and *Actinomyces* were commonly detected as abundant genera (see Additional files 1, 2 and 3 for a detailed summary of abundance rates).

**Fig. 3:**
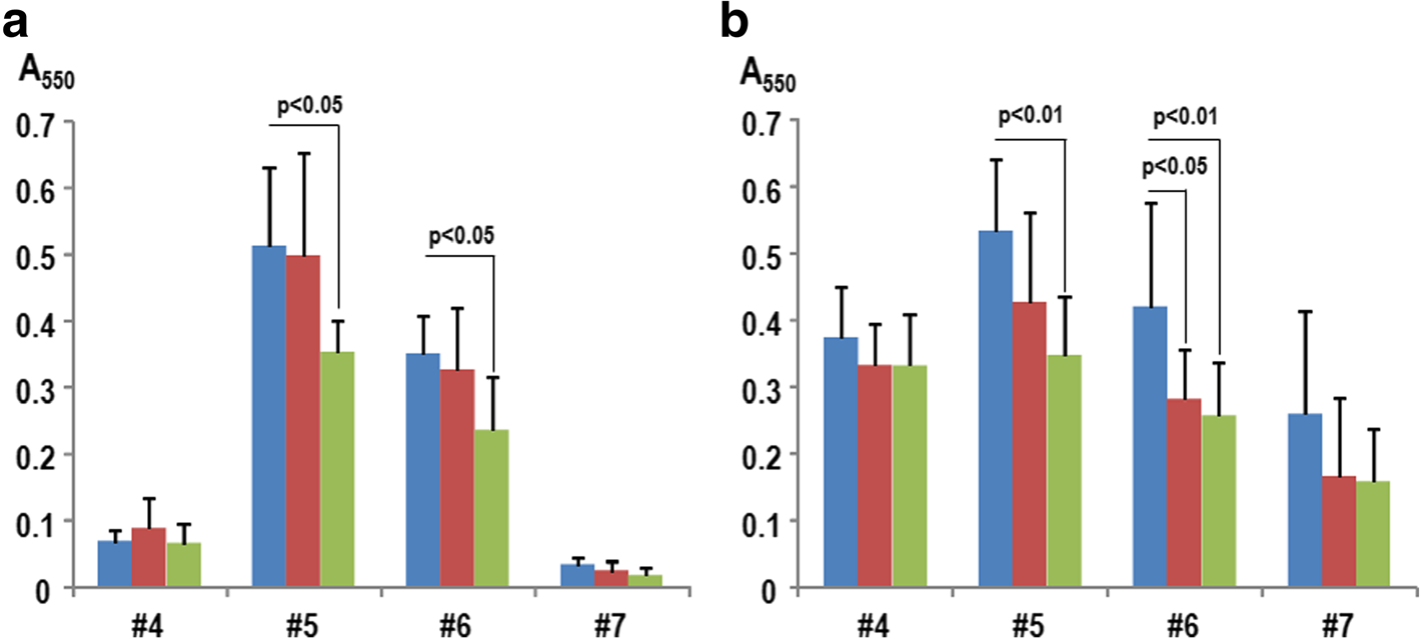
Destabilization of oral biofilms by acidic L-arginine. Cleansing effect of saline, 10 mM citrate (pH3.5) or L-arginine in 10 mM citrate (pH3.5) on established biofilms using human saliva alone (**a**) or human saliva mixed with *S. mutans* GS5 (1 % v/v) (**b**) was examined. The human saliva-derived biofilms were prepared in microwell. After washing with the test reagents, the sustained biofilms were stained with crystal violet. The numbers indicate the IDs of volunteers. Saline, 10 mM citrate (pH3.5) and acidic L-arginine (pH3.5) are indicated by blue, red, and green columns, respectively. Data are expressed as mean ± standard deviation, and the statistical analysis was performed by ANOVA followed by Tukey’s test. The *p*-values less than 0.05 were considered to be significant

The saliva samples were added to microwells containing BHI and 1 % sucrose (20 % v/v), and biofilms were formed following anaerobic incubation for 72 h. Interestingly, the biofilm characteristics differed among samples, wherein the salivary biofilms from two individuals (#5 and #6) tightly attached to the wells (defined as solid biofilms), while the other two (#4 and #7) were easily detached by washing with saline (defined as fragile biofilms) (Fig. 3a). Washing the wells with acidic L-arginine detached more biofilms from #5 and #6 than did saline (*p* < 0.05), whereas 10 mM citrate (pH3.5) alone was similar to that of saline.

To simulate the cariogenic biofilm, *S. mutans* GS5 was added to 1 % of the final well volume. The addition of *S. mutans* GS5 changed the biofilm structure and increased the attachment to the microwells, especially in saliva samples that formed fragile biofilms (Fig. 3 and Additional file 4). Similar to the biofilm cleansing assay without *S. mutans* GS5, acidic L-arginine significantly detached more biofilm derived from the saliva of #5 and #6 (Fig. 3a). In contrast, biofilm containing strain GS5 became sensitive to washing with 10 mM citrate (pH3.5) (Fig. 3b). Meanwhile, acidic L-arginine (pH3.5) and 10 mM citrate (pH3.5) also tended to reduce biofilms formed by fragile biofilm-forming saliva samples (#4 and #6) and *S. mutans* GS5, but no significant differences were observed.

### Biofilm structure after washing with acidic L-arginine

**Fig. 4:**
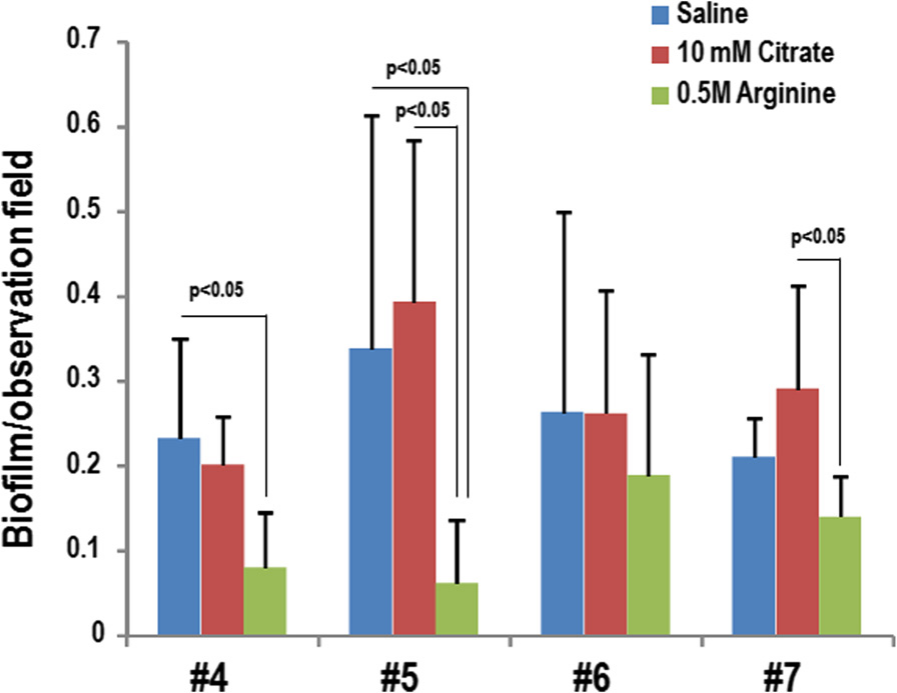
Quantification of sustained oral biofilm after cleansing. Oral biofilms were formed on plastic discs by anaerobic cultivation in BHI broth containing sucrose (1 %) and human saliva (10 %). After a 72 h cultivation at 37 °C, the discs were washed with 1 ml PBS (pH7.4). The discs were further washed with 1 ml saline, 10 mM citrate (pH3.5) or 0.5 M L-arginine (pH3.5) for 30 min before fixing with 2 % glutaraldehyde in 0.2 M cacodylate buffer for scanning electron microscopy. Biofilm areas were measured using a color-based auto-selection tool in Photoshop CS6, and the ratios relative to the total observation field were calculated. At least five randomly selected fields were examined. Data are expressed as mean ± standard deviation, and the statistical analysis was performed by ANOVA followed by Tukey’s test. The *p*-values less than 0.05 were considered to be significant

To further compare the human saliva (#4 to #7)-derived biofilm after washing with saline, citrate (pH3.5), or acidic L-arginine (pH3.5), scanning electron microscopy (SEM) was performed. Biofilm was prepared on a plastic disc using each of the saliva samples and washed with the three test solutions (saline, 10 mM citrate pH3.5, or 0.5 M L-arginine pH3.5). The retaining biofilm area after the washing was quantified at five randomly selected SEM fields by Photoshop CS6 software. As shown in Fig. 4, biofilm mass was reduced to the greatest degree by acidic L-arginine (pH3.5). For samples washed with saline and 10 mM citrate (pH3.5), thick, mat-like objects were retained after washing, whereas acidic L-arginine removed the majority of these structures (Additional file 5).

### Oral bacterial groups susceptible to rinsing with acidic L-arginine

**Fig. 5:**
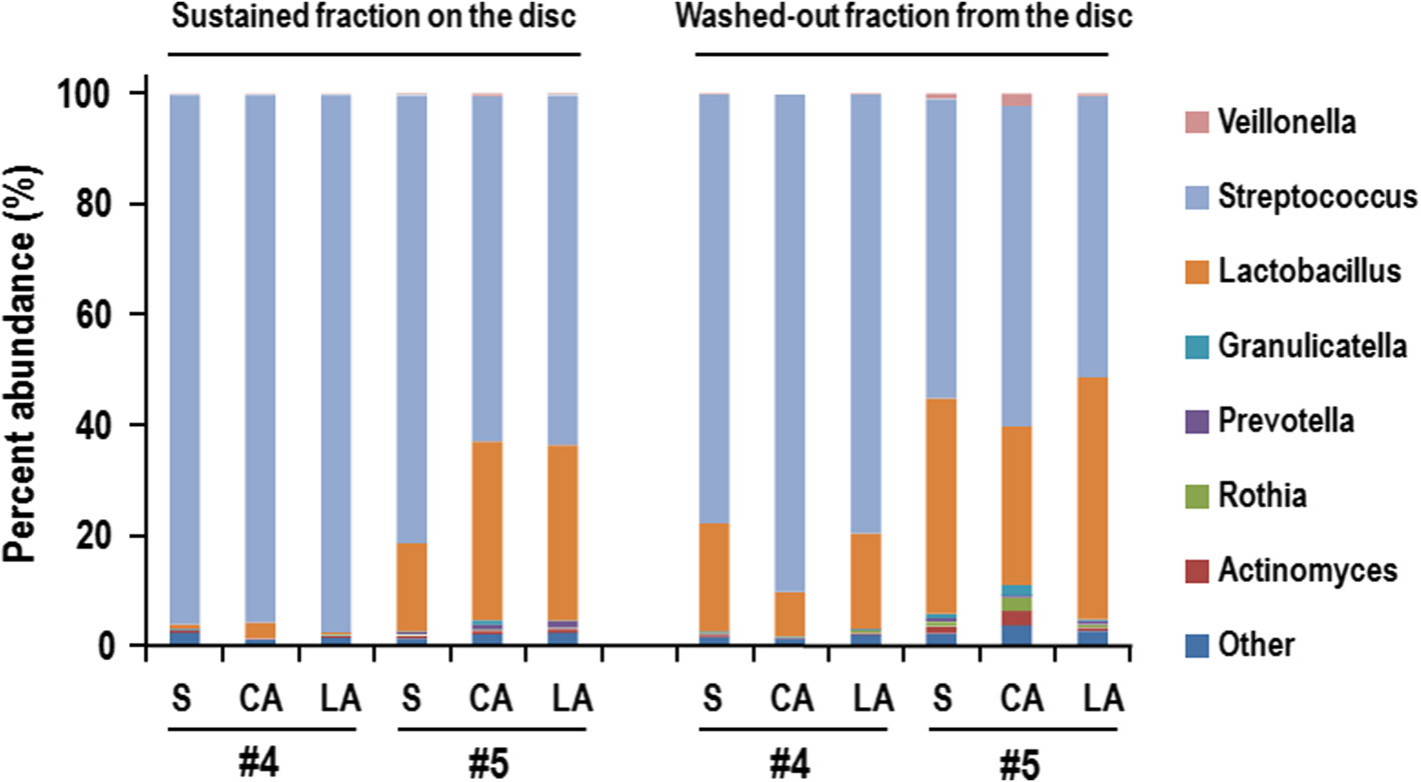
Comparative microbiota analysis of sustained and washed-out fractions with acidic L-arginine. Fragile (from #4) and solid biofilms (#5) were formed on plastic discs, which were then washed with saline (S), 10 mM citrate buffer pH3.5 (CA), or acidic L-arginine (LA). The microbial composition of the fraction remaining on the discs and the washed-out fraction were compared by Illumina-based 16S rDNA sequencing analysis

Illumina-based 16S rDNA sequencing analysis was performed to identify the bacterial groups that were sensitive to rinsing with acidic L-arginine. We tested salivary biofilm from samples #4 and #5 to represent solid and fragile biofilms, respectively. Salivary biofilms were prepared on plastic disc and washed with saline, 10 mM citrate (pH3.5) or 0.5 M L-arginine (pH3.5) for 30 min. The detached biofilms in the test solutions were collected by centrifugation (washed-out fraction). The retaining biofilms on the discs were detached by sonication and collected by centrifugation (retaining fraction). The DNAs were purified from both factions. As shown in Fig. 5, *Streptococcus* and *Lactobacillus* shared 86.8–98.3 % of biofilm populations in both retained and washed-out fractions. Solid biofilm (#5) contained more *Lactobacillus* than fragile biofilm (#4). Interestingly, the *Streptococcus* proportion in the washed-out fraction with citrate buffer (10 mM, pH3.5) was higher than in those with saline or 0.5 M L-arginine (pH3.5). On the other hand, *Lactobacillus* tended to retain on the discs after washing with citrate (pH3.5). These results indicated that citrate (pH3.5) preferentially washed out *Streptococcus* from both biofilm types. On the other hand, the microbial profile of the wash fractions treated with acidic L-arginine was similar to that seen for saline, indicating that acidic L-arginine (pH3.5) non-specifically detached bacteria from biofilms formed on plastic discs.

## Discussion

Arginine deiminase in oral bacteria metabolizes L-arginine that in turn raises the pH of the oral environment, which may suppress the formation of dental caries. Thus, L-arginine has been explored as a potential supplement to oral hygiene care [37]. In addition, high concentrations of L-arginine (5.0–10.0 %) have been reported to inhibit biofilm formation of cariogenic *S. mutans* [26]. In this study, we explored the cleansing effect of L-arginine on oral biofilms to demonstrate the benefits of this basic amino acid for oral hygiene care. L-arginine inhibition of *S. mutans* biofilm formation was indeed pH-dependent (Fig. 1), because among the range of tested pH values (3.5–7.0), significant reductions in biofilm formation were only observed at pH3.5. Therefore, we tested relatively high concentrations (0.5 M) of acidic L-arginine (pH3.5) in this study, and showed the effectiveness of this solution in removing already established oral biofilms. Because L-arginine enhances protein solubility by affecting protein-protein interactions and this effect is evident at acidic conditions [38, 39], the cleansing effect on oral biofilms by acidic L-arginine that was observed here is likely derived from a destabilization of bacterial aggregation.

During the course of this study, Kolderman et al. reported the destabilizing effect of L-arginine on biofilms formed by saliva pooled from six healthy volunteers [40]. This study reported that neutral pH L-arginine (0.1–0.5 M) effectively destabilized oral biofilm. Herein, we showed that acidic pH L-arginine not only destabilized human oral biofilm but also inhibited biofilm formation by *S. mutans*, although it is difficult to compare the results in both studies due to differences in the biofilm assay system used: the Kolderman et al. study used filtered, cell-free saliva as a nutrient, whereas here we used BHI containing 1 % sucrose as the supporting medium for biofilm formation. Sucrose is a cariogenic sugar that enhances the volume and solidity of oral biofilm. The effect of sucrose on in vitro oral biofilm formation was clearly observed in this study, wherein added sucrose apparently altered the biofilm mass and its composition with a marked increase in the number of rod-shaped bacteria (Additional file 4). Considering that *Streptococcus* and *Lactobacillus* were the predominant components of the oral biofilms tested in this study (Fig. 5), these rod-shaped bacteria were likely lactobaclli. Such solid-biofilms formed in rich media may be difficult to remove by neutral L-arginine, and acidic conditions may facilitate destabilization of the aggregates by L-arginine. In fact, Ikeda et al. revealed that the antiviral effect of 0.7 M L-arginine on influenza virus type A was most evident when the pH was below pH5.0 [41].

We selected citrate buffer to adjust the pH of the L-arginine solution because this acid has chelating activity. The inhibitory effect of chelating agents on microbial biofilm is well known, as evidenced by the frequent use of EDTA or citrate in catheter lock solutions. Co-aggregation of *S. mutans* and *Lactobacillus* has been reported to be calcium dependent [42], while tight linkages within *S. mutans* bacteria in *Streptococcus* biofilms mediated by glucan-binding lectins are also dependent on divalent cations [43]. Therefore, citrate would be expected to destabilize biofilms containing *S. mutans* groups. This idea seemed to be supported by the finding that citrate reduced salivary biofilms formed with *S. mutans* GS5, whereas the acid was not effective against biofilms that lacked *S. mutans* (Fig. 3). In addition, Illumina-based analysis of wash fractions from oral biofilms demonstrated that citrate washed out *Streptococcus* to a greater degree than did saline or acidic L-arginine (Fig. 5), indicating that a chelating effect of citrate might be responsible for the removal of streptococci from the biofilm.

The inhibitory and cleansing effects on oral biofilm produced by acidic L-arginine were likely not bactericidal, because acidic L-arginine (pH3.5) reduced *S. mutans* GS5 by only 0.49 ± 0.22 log_10_ CFU during a 5 min contact. In addition, acidic L-arginine reduced the numbers of salivary bacteria from the four healthy volunteers by less than 1.0 log_10_ CFU/ml saliva (data not shown). In their review, Marsh et al. emphasized the importance of oral hygiene regimens that do not kill resident oral microflora, since intact oral microbiota can play a crucial role in maintaining oral health through colonization resistance or modification of oral epithelial cell physiology [44]. The action of L-arginine meets this viewpoint, because this amino acid appears to suppress overgrowth of cariogenic bacteria without massive destruction of normal oral flora. Recently, L-arginine was reported to show an antiviral effect against envelope viruses such as herpes simplex virus and influenza virus [41, 45]. These findings, together with our results indicate that mouth rinses supplemented with an acidic L-arginine solution could inhibit dental plaque formation and also protect against viral infection.

Although L-arginine and citrate are common and safe food components, using acidic L-arginine solution (pH3.5) in a mouthwash could promote enamel demineralization, as sustained exposure to pH < 5.5 induces tooth demineralization and increases the risk of caries [46]. However, since many fruit juices and soft drinks have pH < 3.0 [46], the buffering action of saliva and arginine deiminase systems in oral bacteria may restore oral pH values to safe levels even after the use of a mouthwash with acidic L-arginine (pH3.5). Nonetheless, this point should be investigated in clinical studies to assess the applicability of acidic L-arginine to oral hygiene care.

Notably, the saliva samples from the four healthy volunteers included in this study formed biofilms with different characteristics, i.e., one was solid and the other was fragile. We analyzed the salivary microbiomes of the solid and fragile biofilms (three solid-biofilm formers, SBF; three fragile-biofilm formers, FBF) by Illumina-based 16S rDNA sequencing analysis. A principal coordinate analysis based on a UniFrac analysis at the OTU level did not clearly separate the SBF and LBF samples (Additional file 6), indicating that the different biofilm characteristics might be derived from strain-level variations or differences in host factors. Because the salivary pellicle is a basis for dental plaque formation, the cleansing effect of acidic L-arginine might be influenced by inter-individual variations in host factors such as salivary glycoproteins and mucins, as well as the microbial composition.

## Conclusions

L-arginine has the potential to maintain oral health by elevating dental plaque pH, inhibiting biofilm formation, and destabilizing biofilm structure. In this study we showed that acidic L-arginine (0.5 M) prepared in 10 mM citrate (pH3.5) efficiently removed oral biofilms formed in the presence of rich nutrients. This effect was likely achieved by the suppressive effect of L-arginine toward aggregated organic matter and the chelating effect of citrate. The destabilization of the oral biofilm structure by acidic L-arginine is expected to facilitate washout of dental plaque during routine oral hygiene care, although its effect may vary among individuals. Therefore, future studies are needed to determine which types of oral microbiota or host backgrounds determine L-arginine sensitivity and to design oral hygiene care regimens that use this amino acid. Development of agents that include L-arginine may contribute to the reduction of dental caries, periodontal diseases, or HCAP. For this purpose, the effect of L-arginine on oral microbiota should be carefully evaluated by clinical trials in the future.

## Additional files

Additional file 1. Comparison of OTU (97 % identity) richness derived from rarefaction curves for saliva from healthy volunteers. By analyzing the 20,000 high quality sequences per sample, 300 to 400 operational taxonomy units (OTUs, > 97 % identity) were detected in the samples. (TIF 645 kb) Additional file 2. Microbial community structures in saliva from healthy volunteers. *Streptococcus* and *Prevotella* represented more than 5 % of the total salivary microbiota in all of the samples tested. See Additional file 3 for detailed information on the relative abundance of each genus. The salivary microbiota composition at the genus level was similar among the samples. The Shannon-Weaver indices at the genus level of the salivary microbiome from samples #4, #5, #6, and #7 were 2.92, 2.75, 2.64, and 2.29, respectively. (TIF 1230 kb) Additional file 3. Summary of percent abundance of salivary microbiota from healthy volunteers. (XLS 48 kb) Additional file 4. Effect of 1 % sucrose and exogenously added *Streptococcus mutans* on biofilm formation by salivary bacteria. Human saliva was cultured anaerobically for 72 h at 37 °C in BHI only (a, d), or BHI containing 1 % sucrose without (b, e) or with (c, f) 1 % volume of *S. mutans* (OD_600_ = 0.7). The biofilm formed on plastic discs was examined by scanning electron microscopy at low (500x, panels a, b, c) and high (3,000x, panels d, e, f) magnification. Sucrose enhanced biofilm formation by human salivary bacteria. Culturing with sucrose decreased filamentous-form bacteria (indicated by white arrowheads) but increased the number of rod-shaped bacteria. Extracellular polysaccharide-like objects (white arrows) were observed when the sucrose solution was supplemented with *S. mutans.* Bars in upper and lower panels indicate 50 μm and 10 μm, respectively. (TIF 592 kb) Additional file 5. Scanning electron microscopy of oral biofilms after cleansing. Among the selected fields at 100x magnification, the most biofilm-rich area in each sample is shown. Thick biofilm were observed even after washing with saline or 10 mM citrate (pH3.5), whereas these thick biofilms were removed after washing with acidic L-arginine (pH3.5). Bars indicate 200 μm. (TIF 572 kb) Additional file 6. PCoA plot describing unweighted UniFrac distance between salivary samples. Pairwise distances between all samples are projected onto a two dimensional space where the PCA axis describes the highest degree of variation. Samples that are clustered closely together are thus considered to share a larger proportion of the phylogenetic tree compared to samples that have a larger separation. (TIF 47 kb)
