# Supplementary figures and images for: Cleansing effect of acidic L-arginine on human oral biofilm
Source: BMC Oral Health. 2016 Mar 22;16:40. doi: 10.1186/s12903-016-0194-z (PMC4802732; doi:10.1186/s12903-016-0194-z)

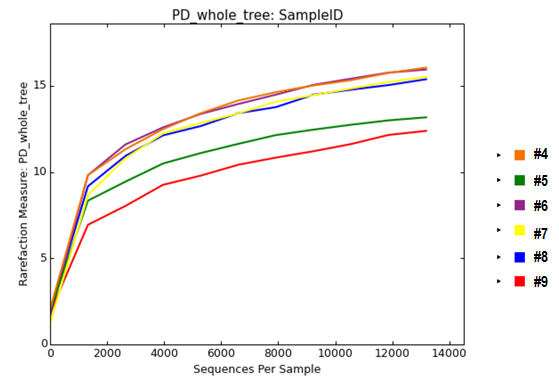

Supplement: Additional file 1. — Comparison of OTU (97 % identity) richness derived from rarefaction curves for saliva from healthy volunteers. By analyzing the 20,000 high quality sequences per sample, 300 to 400 operational taxonomy units (OTUs, > 97 % identity) were detected in the samples. (TIF 645 kb) [file 12903_2016_194_MOESM1_ESM.tif]

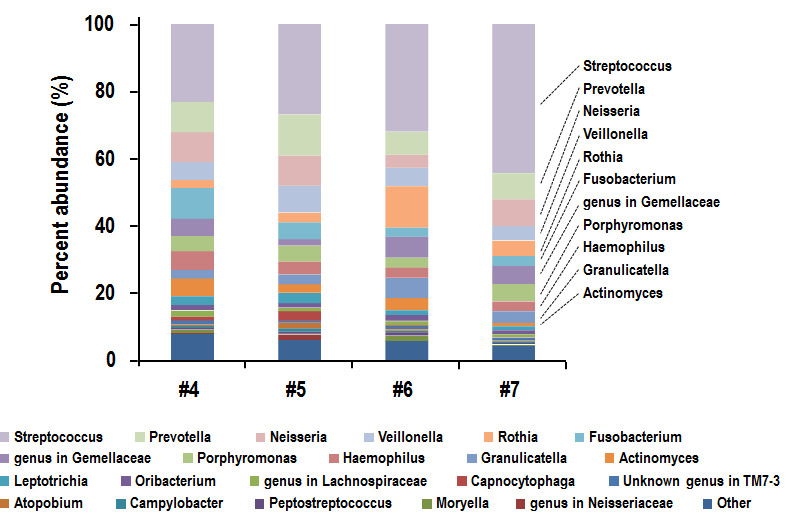

Supplement: Additional file 2. — Microbial community structures in saliva from healthy volunteers. Streptococcus and Prevotella represented more than 5 % of the total salivary microbiota in all of the samples tested. See Additional file 3 for detailed information on the relative abundance of each genus. The salivary microbiota composition at the genus level was similar among the samples. The Shannon-Weaver indices at the genus level of the salivary microbiome from samples #4, #5, #6, and #7 were 2.92, 2.75, 2.64, and 2.29, respectively. (TIF 1230 kb) [file 12903_2016_194_MOESM2_ESM.tif]

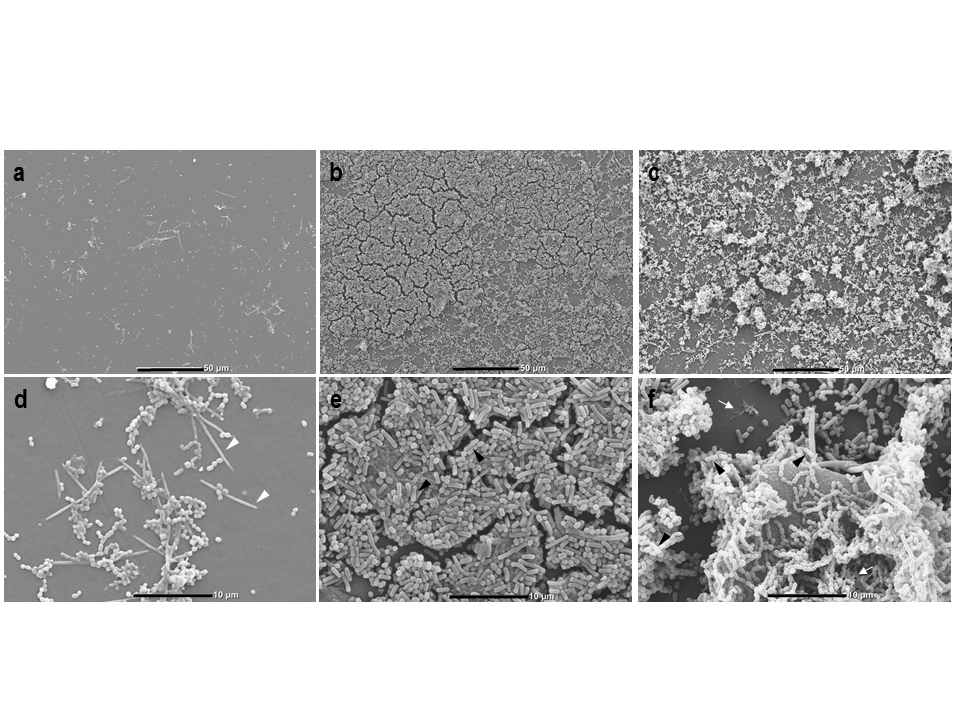

Supplement: Additional file 4. — Effect of 1 % sucrose and exogenously added Streptococcus mutans on biofilm formation by salivary bacteria. Human saliva was cultured anaerobically for 72 h at 37 °C in BHI only (a, d), or BHI containing 1 % sucrose without (b, e) or with (c, f) 1 % volume of S. mutans (OD600 = 0.7). The biofilm formed on plastic discs was examined by scanning electron microscopy at low (500x, panels a, b, c) and high (3,000x, panels d, e, f) magnification. Sucrose enhanced biofilm formation by human salivary bacteria. Culturing with sucrose decreased filamentous-form bacteria (indicated by white arrowheads) but increased the number of rod-shaped bacteria. Extracellular polysaccharide-like objects (white arrows) were observed when the sucrose solution was supplemented with S. mutans. Bars in upper and lower panels indicate 50 μm and 10 μm, respectively. (TIF 592 kb) [file 12903_2016_194_MOESM4_ESM.tif]

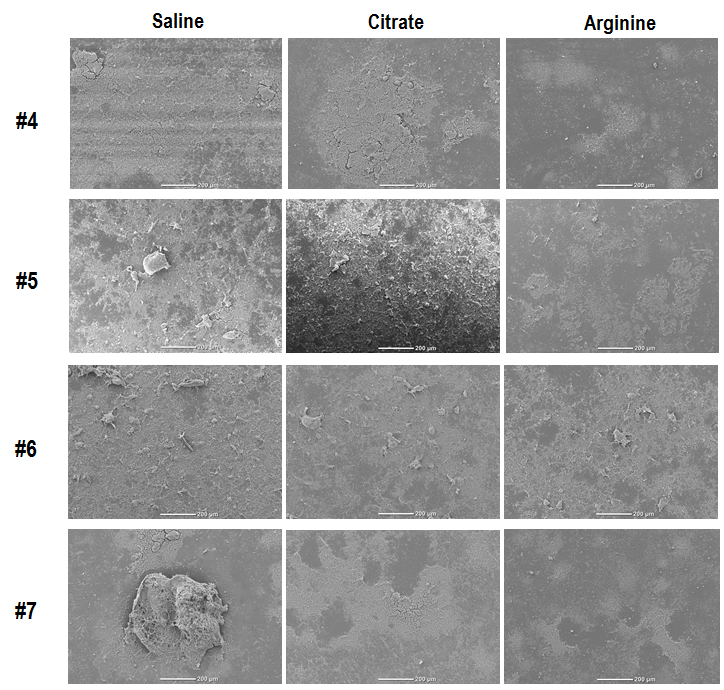

Supplement: Additional file 5. — Scanning electron microscopy of oral biofilms after cleansing. Among the selected fields at 100x magnification, the most biofilm-rich area in each sample is shown. Thick biofilm were observed even after washing with saline or 10 mM citrate (pH3.5), whereas these thick biofilms were removed after washing with acidic L-arginine (pH3.5). Bars indicate 200 μm. (TIF 572 kb) [file 12903_2016_194_MOESM5_ESM.tif]

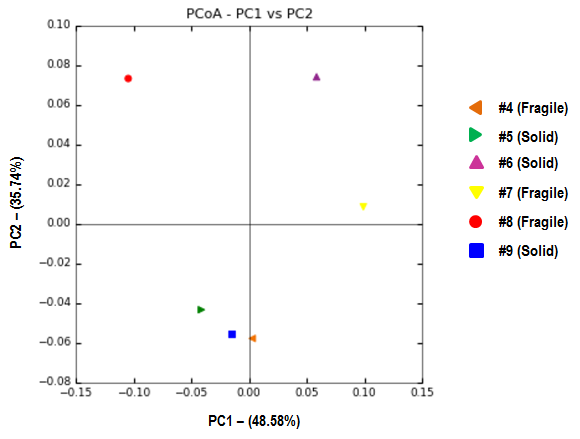

Supplement: Additional file 6. — PCoA plot describing unweighted UniFrac distance between salivary samples. Pairwise distances between all samples are projected onto a two dimensional space where the PCA axis describes the highest degree of variation. Samples that are clustered closely together are thus considered to share a larger proportion of the phylogenetic tree compared to samples that have a larger separation. (TIF 47 kb) [file 12903_2016_194_MOESM6_ESM.tif]
